# Enhanced porphyrin-based hypoxia imaging by temporal oversampling of delayed fluorescence signal

**DOI:** 10.1117/1.JBO.30.S2.S23903

**Published:** 2025-01-28

**Authors:** Marien I. Ochoa, Arthur F. Petusseau, Matthew S. Reed, Petr Brůža, Brian W. Pogue

**Affiliations:** a University of Wisconsin–Madison, Department of Medical Physics, Madison, Wisconsin, United States; b Dartmouth College, Thayer School of Engineering, Center for Imaging Medicine, Hanover, New Hampshire, United States

**Keywords:** hypoxia imaging, protoporphyrin IX, porphyrins, functional imaging, bio-luminescence imaging, lifetime imaging

## Abstract

**Significance:**

Protoporphyrin IX (PpIX) delayed fluorescence (DF) is inversely related to the oxygen present in tissues and has potential as a novel biomarker for surgical guidance and real-time tissue metabolism assessment. Despite the unique promise of this technique, its successful clinical translation is limited by the low intensity emitted.

**Aim:**

We developed a systematic study of ways to increase the PpIX DF signal through acquisition sampling changes, allowing optimized imaging at video rates.

**Approach:**

To accomplish signal increase, time-gating signal compression was achieved through changes in pulse frequency and power density, using sampling rates that are faster than the decay rate of the signal. The increased signal yield was tested and validated *in vitro* and then demonstrated *in vivo*, with comparison to settings that sample the full lifetime emission decay.

**Results:**

Results *in vitro* and *in vivo* demonstrated that optimized timing could increase the detected intensity by a factor of 7. The images showed results that were superior than when sampling the full DF lifetime decay.

**Conclusions:**

The proposed timing optimization enhances PpIX-based DF real-time imaging of tissue hypoxia. By increasing sampling frequency and adjusting the acquisition gate and pulse width, the collected signal intensity improved sevenfold, demonstrated both *in vitro* and *in vivo*. The technique was shown to enable better visualization of small and anatomically challenging hypoxic structures. The improved target-to-background ratio and compatibility with pressure-enhanced sensing of tissue oxygen technique were demonstrated.

## 1 Introduction

Understanding tissue oxygen content, especially the lack of oxygen, is key for assessing proper tissue function. Of special interest are tumors, which often grow with chaotic neo-vasculature, subsequently creating both chronic and transient hypoxia conditions. The administration of 5-ALA^1^^,^^2^ as a precursor of protoporphyrin IX (PpIX) is widely adopted in neurosurgery to guide glioma resection,^1^^,^^3^ as well as in photodynamic therapy (PDT).^4^^–^^6^ Although nearly all PpIX imaging applications capture the prompt fluorescence (PF) component of PpIX, a more rarely explored property of PpIX is its weaker delayed fluorescence (DF) component.^7^^–^^9^ The DF component^10^ of PpIX arises from emissions originating from the first excited singlet state, which has been replenished through reverse intersystem crossing from the excited triplet state where oxygen is significantly quenched. Investigations of PpIX DF imaging as a marker of hypoxia have been reported for surgical guidance,^8^^,^^11^^,^^12^ PDT,^7^ and burn injuries^13^^,^^14^ where the DF component is isolated using time-gated cameras. From a biology standpoint, the use of techniques such as pressure-enhanced sensing of tissue oxygen (PRESTO)^11^^,^^12^ where tissue is palpated to create transient hypoxia and hence increase DF signal output has been proposed. However, the PRESTO effect can vary depending on the application; therefore, finding a complementary solution that can globally increase DF signal output is desired. Despite the utility of the technique to obtain real-time imaging of hypoxia, PpIX DF intensity is orders of magnitude lower than the standard PF, and this is why it has been largely ignored. Detection of this signal becomes a challenge as external light sources (e.g., room lights) can overwhelm the signal and make it inapplicable to open surgery settings where room lights are needed. Even when the white light is time-gated, aiding to see the FOV, achieving the minimum level of background light required for DF imaging remains a limitation. In this paper, the ability to increase the DF intensity and maximize signal gathering by variation of both excitation and detection time windows is explored. Of importance is the speed of acquisition to image fast oxygen kinetics in real time. A key driver in this work was the realization that recovery of the full-emission lifetime^15^ decay was likely not required, but rather, simply capturing the maximum signal intensity of the DF signal was the goal. This realization steered this work toward overdriving the frequency of capture, to maximize DF emission signal collection immediately after the excitation pulse. We achieved this using a faster frequency to overdrive the sampling rate as well as optimized pulse-width and gate-width settings. This technical advancement was explored here as a way to substantially increase the signal-to-background ratio of the images while keeping real-time frame rates.

## 2 Methods

**Fig. 1:**
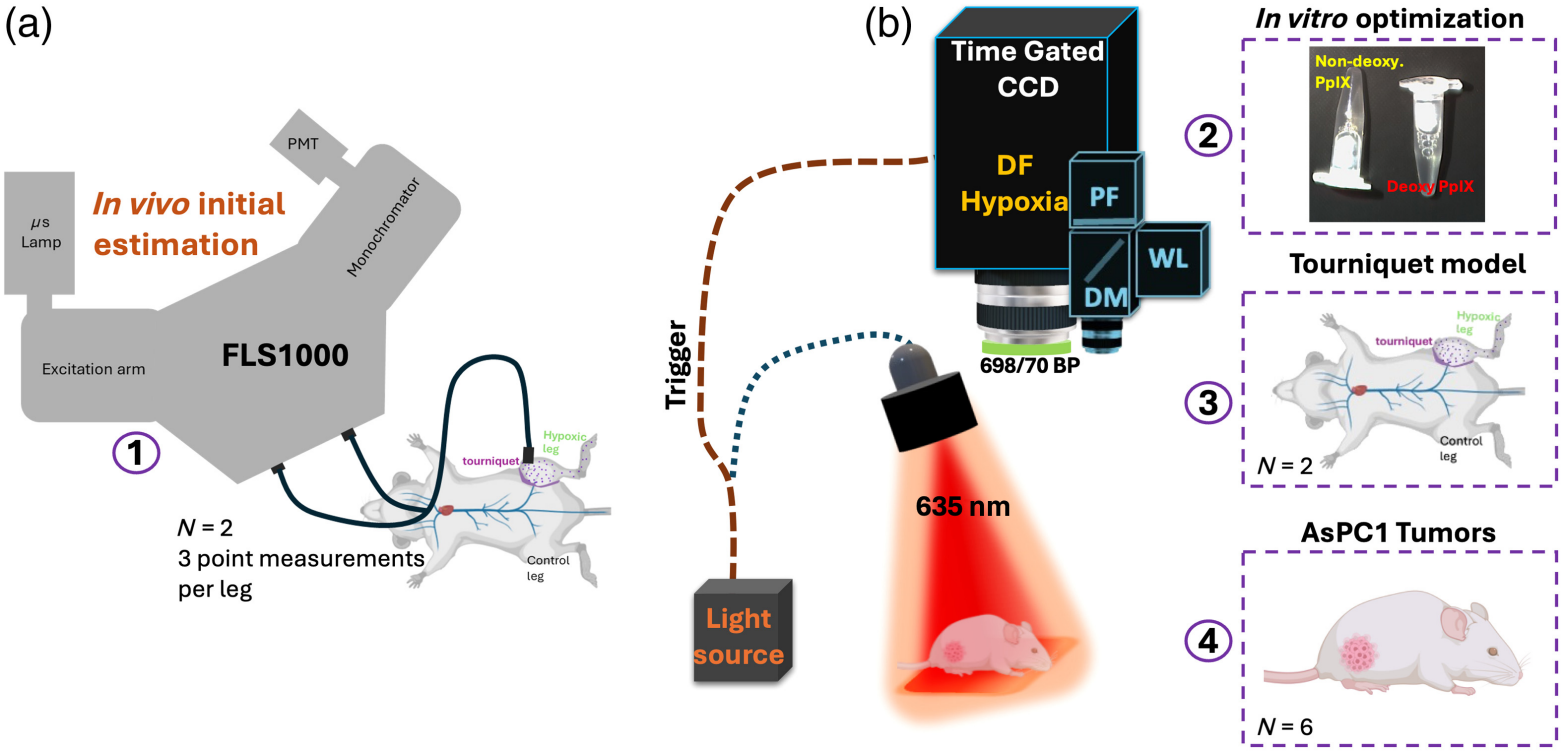
Graphical description of used instruments and methods. (a) Depiction of FLS1000 fiber-based commercial system. FLS1000 used for initial *in vivo* estimation of lifetime decay length and comparison of DF/PF spectra. (b) Depiction of setup for macroscopic imaging of PpIX PF and DF/Hypoxia. Numbers 1 to 4 describe the order in which experiments were executed. 1. Preliminary estimation of DF spectrum and lifetime decay length *in vivo* through fiber-based commercial instrument. 2. *In vitro* optimization of temporal oversampling settings for PpIX hypoxia imager. 3. Validation of temporal oversampling settings for the same *in vivo* model used in 1. 4. Validation of temporal oversampling settings for AsPC1 tumors. Used number of mice *N* also indicated when applicable.

The workflow of experiments is depicted in Fig. 1. This work used two instruments: the FLS1000 (fiber-attachment) time-gated commercial spectrometer and a custom-made time-gated setup for macroscopic imaging of PpIX hypoxia. FLS1000 acquires single-point spectral and time-domain fluorescence measurements, whereas the PpIX setup acquires 1024×1024  pixel images at a maximum frame rate of 40 fps. The FLS1000 was only used to have an initial estimate of the length of the fluorescence lifetime decay of PpIX DF *in vivo*. This information was then used to estimate gate widths of interest for *in vivo* imaging with the PpIX hypoxia setup. As the FLS1000 is also a spectrometer, it was used to compare the spectrum of both PpIX PF and DF. Even though the FLS1000 was used for initial estimation of PpIX PF/DF spectra and lifetime decay length, the main goal of this work was the optimization of the PpIX imager and its time-domain parameters for enhanced *in vivo* mapping of hypoxia. For the PpIX hypoxia imager and to minimize changes that could arise from *in vivo* conditions, optimization was first done *in vitro*, and optimal settings were subsequently validated for *in vivo* murine models of hypoxia (tourniquet and AsPC1 tumors).

### 2.1 Initial Estimation of Spectral and Temporal Behavior of PpIX DF *In Vivo* with Commercial Spectrometer

Before choosing target temporal sampling settings for the hypoxia wide-field imager, a commercial photoluminescence spectrometer (FLS1000, Edinburgh Instruments, Livingston, United Kingdom) was used to estimate the temporal behavior of the PpIX DF signal *in vivo*. The commercial instrument is depicted in Fig. 1(a). An *in vivo*–controlled model of hypoxia was created via a tourniquet that was applied to the right leg of a mouse and secured. The tourniquet on the hind limb was used as a simple but effective method to stop blood circulation to the tissue, thereby inducing deep hypoxia in the targeted area. For initial estimation, a mouse model of hypoxia was preferred as *in vivo* imaging was the end goal. The photoluminescence spectrometer was used to characterize the spectrum of both PF and DF *in vivo* signals from 610 to 710 nm. The lifetime measurement was performed through the lifetime imaging setting with time steps of 10  μs and 635 nm excitation at a frequency of 10 Hz. PF signals were collected at the same time as the excitation pulse, whereas DF signals were acquired by time gating to collect after the illumination pulse. Spectrometer measurements were made using a fiber-based attachment provided with the instrument [Fig. 1(a)]. The probe was scanned in three different locations for the hypoxic leg (with tourniquet) and for the control leg. This process was repeated for N=2 mice. As the instrument could not provide an image, it was only used to verify two key aspects: whether the emission spectra of both PpIX DF and PF were the same and to have an initial estimation of the full lifetime decay of DF. This preliminary information was expected to offer insights into potential target frequencies and pulse width settings to optimize the wide-field hypoxia imager. In addition, a similar PF and DF spectrum would confirm that the same bandpass filter could be used to isolate both PF and DF signals.

### 2.2 Optimization of PpIX Hypoxia Imager Through Temporal Oversampling

#### 2.2.1 PpIX hypoxia imager

To investigate PpIX-based hypoxia’s macroscopic distribution, a previously described setup^9^^,^^13^ was used. The optical arrangement is depicted in Fig. 1(b). The sample plane was illuminated using a 635-nm pulsed LED (SOLIS-620D, Thorlabs, Newton, New Jersey, United States), coupled with a 635-nm band-pass filter (FF01-698/70-25, Semrock, Rochester, New York, United States), covering a $6\times 6\;{\mathrm{cm}}^{2}$ field of view (FOV). The illumination source can be adjusted for pulse width, pulse frequency, and power output, with a maximum continuous wave (CW) power output of 4.7 W. These three parameters were varied for the purpose of this study. For the collection of DF signals, the setup employed a time-gated^16^^–^^18^ intensified charge-coupled device (emICCD) camera (Pimax 4, Teledyne Princeton Instruments, Inc., Trenton, New Jersey, United States), synchronized to capture time-domain information in relation to each illumination pulse and utilizing a gate width in the μs time range.^19^ A 650 long-pass filter was used to eliminate the excitation light. Time-gated collection after the illumination pulse was representative of hypoxia, as shown in Figs. 3(a) and 3(b). In addition, a dichroic mirror, paired with two CMOS sensors (FLIR, Blackfly, Teledyne Technologies, Thousand Oaks, California, United States), concurrently captured white-light and PF images at the same time stamps as the DF channel. For PF, identical filter specifications to those in the DF channel were employed. This setup allowed for real-time imaging of white light (RGB), PpIX PF, and DF signals with minimal image allocation/processing time. PF, acquired during the excitation pulse, provides information on the distribution and concentration of PpIX. On the other hand, DF provides oxygen content (hypoxia) information. Initial testing was performed using *in vitro* models of deoxygenated PpIX solution. This was done to minimize changes in PpIX concentration and oxygen content that might arise from PpIX kinetics *in vivo*, ensuring that any observed results were attributed to changes in the acquisition parameters rather than biological factors.

#### 2.2.2 Preparation of *in vitro* samples

PpIX *in vitro* samples used throughout this study were prepared using dissolved PpIX (Sigma-Aldrich, St. Louis, Missouri, United States) under two conditions: oxygenated and deoxygenated. The PpIX vials are depicted in Fig. 1(b) in box 2. Oxygenated samples were prepared by diluting a 1000-μM PpIX stock solution in a solution of 2% Tween 20 in phosphate-buffered saline (PBS), resulting in a final concentration of 2.5  μM PpIX and a total volume of 500  μL. On the other hand, deoxygenated samples were prepared the same way and deoxygenated using a combination of PBS (465  μL), Tween 20 (10  μL), glucose (10  μL), catalase (5  μL), and glucose oxidase (5  μL) enzymes matching the overall solvent volume of the non-deoxygenated sample.^7^^,^^20^ Solutions were prepared in 500-μL microcentrifuge tubes, and deoxygenated vials were immediately cap-sealed after enzymes were introduced. Both samples were mixed through a vortex mixing instrument. Samples were prepared 20 min before imaging to allow for proper enzymatic activity in the deoxygenated vials.^21^

#### 2.2.3 Method to overdrive photon sampling through frequency increase for *in vitro* samples

To test the effect of frequency variation alone on intensity gain, the power density was adjusted to be approximately constant. A 60-μs pulse was used for excitation. The acquisition window width and the pulse repetition rate were varied respectively as follows: 1800  μs for a 500-Hz frequency, 900  μs for a 1000-Hz frequency, 475  μs for a 2-kHZ frequency, and 225  μs for a 4-kHZ frequency. The total exposure time per acquired frame was maintained at ∼280  ms across all acquisitions. The power density at the imaging plane was set to $0.32\;\mathrm{mW}/{\mathrm{cm}}^{2}$ and was maintained across settings by respectively changing the percentage illumination power. Delay (d) after pulse was kept constant at a value of 10  μs across all tests. This delay ensured that the LED’s post-pulse emission was not considered in the DF measurement. *In vitro* samples were measured at pulsed illumination frequencies of 500, 1000, 2000, and 4000 Hz. The used excitation pulse-width and gate-width settings are illustrated in Fig. 3.

#### 2.2.4 Method to test for signal amplification through increased excitation pulse-width for *in vitro* samples

To understand the role of power density in the DF output signal, the best frequency as determined on the previous test was used, and power density values varied. To efficiently increase power density without the need for higher excitation power (e.g., a different light source), the pulse-width of the excitation pulse was varied. This allowed for efficient re-distribution of power output per pulse. Excitation pulse-widths of 14, 30, 45, 60, 75, and 90  μs were tested. To test pulse-width/power-density effects on the PpIX DF signal intensity, a constant gate-width of 475  μs was used. This was determined from the initial test where frequency was varied while maintaining a constant pulse width and power density.

#### 2.2.5 Influence of longer excitation pulse width and shorter gate width on signal amplification for *in vitro* samples

The goal of this test was to understand the importance of pulse width and power density in relation to the acquisition gate-width size. The initial hypothesis was that a larger acquisition gate width (even with a short pulse width) would result in a higher number of collected photons. To test this, various pulse-width/gate-width combinations were evaluated for values of 90/475  μs, 125/350  μs, 175/300  μs, 225/250  μs, 275/200  μs, 325/150  μs, and 375/100  μs.

#### 2.2.6 *In vivo* validation of *in vitro* estimated optimal settings for the PpIX hypoxia imager

The optimal settings selected from *in vitro* experiments were validated *in vivo* using Athymic nude mice (Envigo/Inotiv, Inc., Lafayette, Indiana, United States) for two different scenarios. First, a tourniquet was applied to the right leg of a mouse with no tumors. Figure 6(a) depicts the target leg, where a string was tightened and locked in place until the skin in the leg appeared blue/purple (in comparison to the control leg) indicating a lack of oxygen. The results were compared for settings where the majority of the PpIX DF lifetime was sampled, to the optimized settings resulting from *in vitro* quantification.

Second, mice with pancreatic adenocarcinoma (AsPC1) tumors were used. AsPC1 pancreatic adenocarcinoma cells were obtained from ATCC and cultured in RPMI 1640 media supplemented with 10% fetal bovine serum and 1% penicillin/streptomycin. Cells were grown in a 5% ${\mathrm{CO}}_{2}$ incubator at 37°C. Cells were harvested and resuspended in a 50% Matrigel/50% PBS mixture. Nude mice (6 to 8 weeks of age, Envigo) received injections into the flank of the right leg at a concentration of $10^{6}$ cells in a 0.1-mL injection. Mice (N=6) were imaged once the tumors reached 5 to 7 mm in diameter (∼3 to 4 weeks after). For imaging in tumors, settings where the majority of the PpIX DF lifetime was sampled were compared with those with optimized settings resulting from *in vitro* quantification [Fig. 7(d)]. In addition, tumors were imaged upon palpation, creating a transient hypoxia effect (PRESTO) as previously reported.^11^^,^^12^

### 2.3 Anesthesia and 5-ALA Administration Procedures

All mice used throughout this work were individually anesthetized (SomnoSuite Low-Flow, Kent Scientific, Torrington, Connecticut, United States) with an initial induction flow rate set to 3% isoflurane in an induction chamber. Then, the flow rate was adjusted to 2% to 2.5% for maintenance under a nose cone. The carrier gas was 100% oxygen at a flow rate of 500 mL/min. Mice were constantly monitored to verify proper breathing patterns. 5-ALA was administered intravenously through retro-orbital injection at a 250 mg/kg dose. Imaging was then conducted under anesthesia at 1 h post-5ALA injection for tourniquet models and 6 h post-5ALA for AsPC1 tumors. This dose was a human-equivalent dose based on $\mathrm{mg}/m^{2}$ conversion of humans to mice, with the estimated dose equivalent value in mice being ∼12.3 times greater than that used in humans when measured by mg/kg dosing.^22^ In compliance with IACUC protocol, mice were housed at UW-Madison vivarium and received a reduced-chlorophyll diet until 2 days prior to imaging, after which they received a chlorophyll-free purified diet (TD.97184, Envigo RMS LLC) to maximally remove autofluorescence from the colon, feces, and skin surface.

## 3 Results

### 3.1 Results for Initial Estimation of Spectral and Temporal Behavior of PpIX DF *In Vivo* With Commercial Spectrometer

**Fig. 2:**
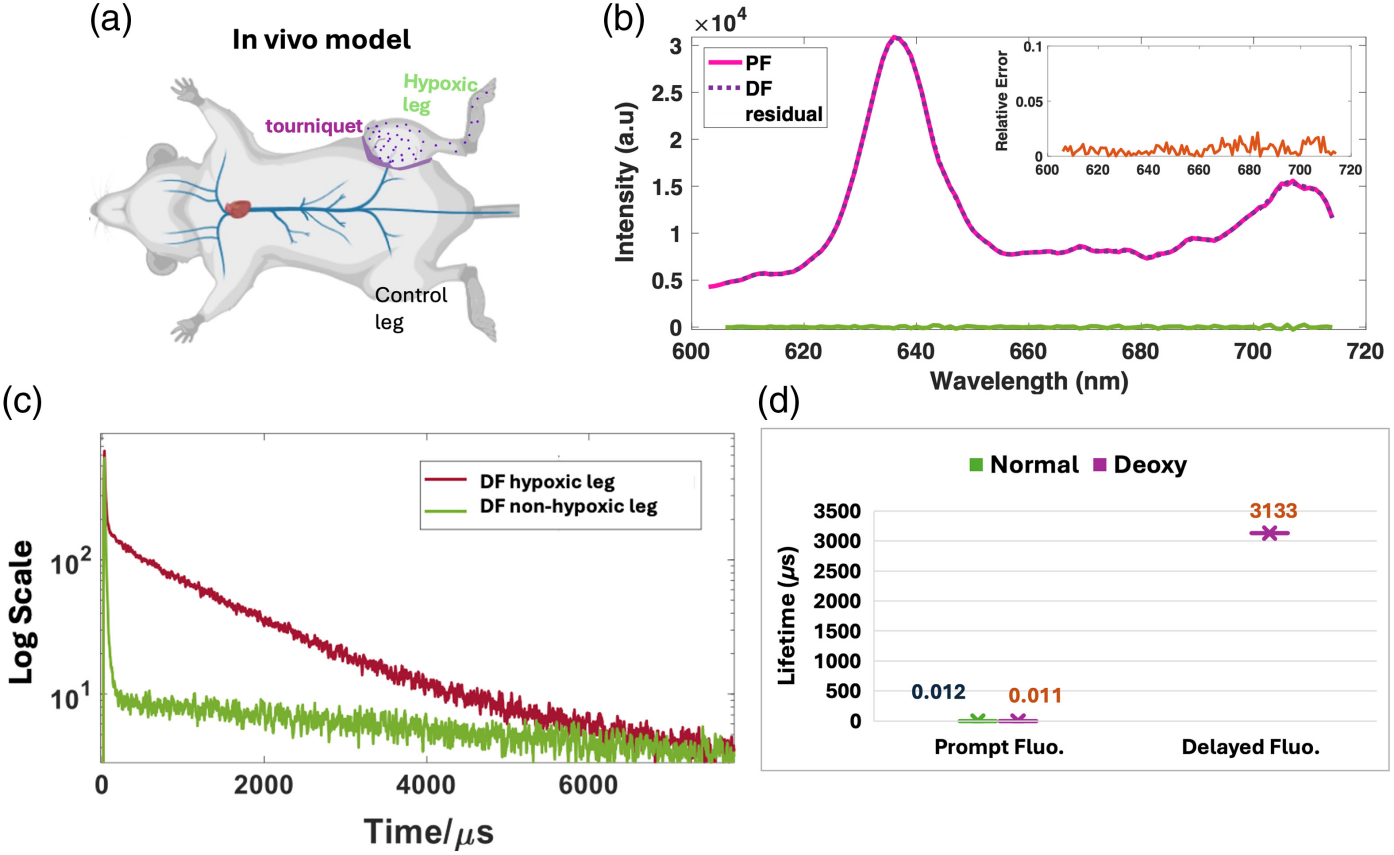
Results for mouse model with induced hypoxia on the right leg. (a) Schematic showing the placement of the tourniquet on the right leg of the murine model. (b) Spectrum of PpIX Prompt versus DF. The residual from DF to PF spectra is shown. Relative error was also estimated. (c) Measured Lifetime decay of PpIX DF on hypoxic leg (with tourniquet) versus normoxic leg. (d) Lifetime quantification in both normoxic and hypoxic conditions, for both PF and DF. DF lifetime in the normoxic case is not displayed because the DF component is negligible.

To gain a deeper understanding of the temporal behavior of the PpIX DF signal indicative of hypoxia, we utilized a basic *in vivo* hypoxia model, applying a tourniquet to the right leg of a mouse to imply blood flow stasis. Through the photoluminescence spectrometer FLS1000 (Edinburgh Instruments Ltd.), the spectra of both prompt (PF) and DF were acquired and are displayed in Fig. 2(b). In addition, the full lifetime decay of the PpIX DF signal in the hypoxic leg was measured and is presented in Fig. 2(c), alongside the PF lifetime decay for comparison. The experimental setup is depicted in Fig. 1(a) and described in Sec. 2.1. The results in Fig. 2(b) display both PF and DF emission spectra. Differences between them were negligible, as shown by the residual values calculated from the subtraction of both spectra. In addition to the residual, the relative error was calculated by

$$\frac{\mathrm{DF}-\mathrm{PF}}{\mathrm{PF}},$$ (1)

where the inferred value was the spectrum of PpIX DF and the true value was assigned to the PF spectrum of PpIX. Figure 2(b) displays the relative error plot where values were below 0.1. Figure 2(c) displays the *in vivo* lifetime decay as measured in mouse leg with the tourniquet. The lifetime was subsequently quantified through a bi-exponential fitting model (propietary software on the FLS1000) and compared with the lifetime values of the PF component. Quantification results are displayed in Fig. 2(d) where the DF lifetime approximates 3133  μs, whereas PF is near 12 ns. The DF component was only observable in hypoxic tissue; hence, why in the case of normoxia, DF lifetime quantification was negligible as there was no DF component. These nanosecond PF lifetimes are consistent with values previously reported,^23^^,^^24^ whereas the DF lifetime is greater by a factor of ∼284,818, as seen in Fig. 2(d).

### 3.2 Results for Overdrive of Photon Sampling Through Frequency Increase for *In Vitro* Samples

Figure 2(c) displays a DF lifetime of 3133  μs, where a window of ∼100,000  μs and a frequency of 10 Hz were needed to collect the full DF lifetime decay. It can be inferred that collecting the full decay window will result in the best signal output. However, the use of higher sampling frequencies ≥500  Hz was considered here. Hence, the acquisition gate width was reduced as a function of frequency increase to prove whether sampling after the excitation pulse and closer to the start of the decay, which is richer in photon counts, could be more beneficial than collecting the full DF lifetime decay.

**Fig. 3:**
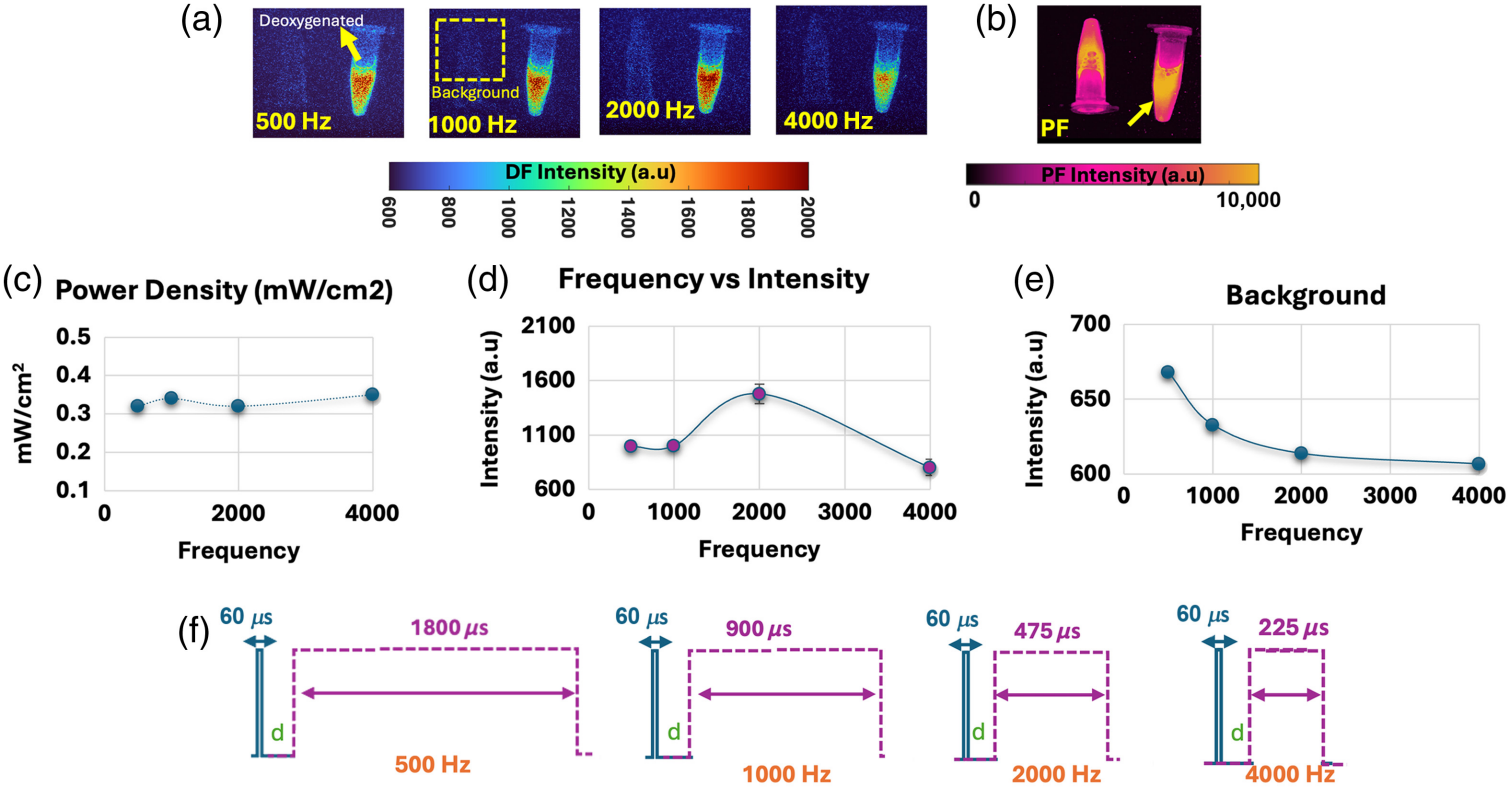
Results for the *in vitro* model of frequency change. Power density, intensity, and background plots are provided for each of the measured settings. (a) Representative frames per each setting. (b) Representative PF image. (c) Power density kept constant to test only frequency-related effects. (d) DF intensity as a function of frequency. (e) Background intensity as a function of frequency. (f) Schematic of used settings. Delay—*d* after each pulse is kept consistent with a value of 10  μs for all used settings.

Figure 3 shows the results obtained across different frequencies for *in vitro* samples of PpIX as excited with constant power and a 60-μs pulse-width and constant power density of $0.32\;\mathrm{mW}/{\mathrm{cm}}^{2}$. Depiction of the *in vitro* samples is shown in Fig. 1(b) box 2, and the preparation is described in Sec. 2.2.2. Figure 3(c) displays the recorded power per unit area with an approximate power density of $0.32\;\mathrm{mW}/{\mathrm{cm}}^{2}$ maintained across settings. Figure 3(a) displays example frames recorded utilizing a gate width of 1800  μs at a 500-Hz frequency, 900  μs at a 1000-Hz frequency, 475  μs at a 2-kHZ frequency, and 225  μs at a 4-kHZ frequency. These excitation pulse-width and gate-width settings are illustrated in Fig. 3(f). As the frequency increases, the usable acquisition window is shortened as there is less available time to be used in the gate width per pulse. The results as quantified in Fig. 3(d) show how PpIX DF is increased by a factor of 1.3 per frame when using a frequency of 2 kHZ, an average of 400 counts more per frame. In addition, as quantified in Fig. 3(e), a slight decrease in background was observed as excitation frequency increased and gate width decreased. Background values through this paper were estimated for *in vitro* samples based on vials that are not de-oxygenated through enzymes, which are displayed on the PF images [Fig. 3(a)] but not on the DF images. When comparing the hypoxia image in Fig. 3(a) at 500 Hz versus its PF image in Fig. 3(b), it is appreciated how the DF channel only showed the deoxygenated vial, whereas the PF fluorescence channel does not differentiate between oxygenated and deoxygenated vials.

### 3.3 Results for Signal Amplification Through Increased Excitation Pulse Width for *In Vitro* Samples

**Fig. 4:**
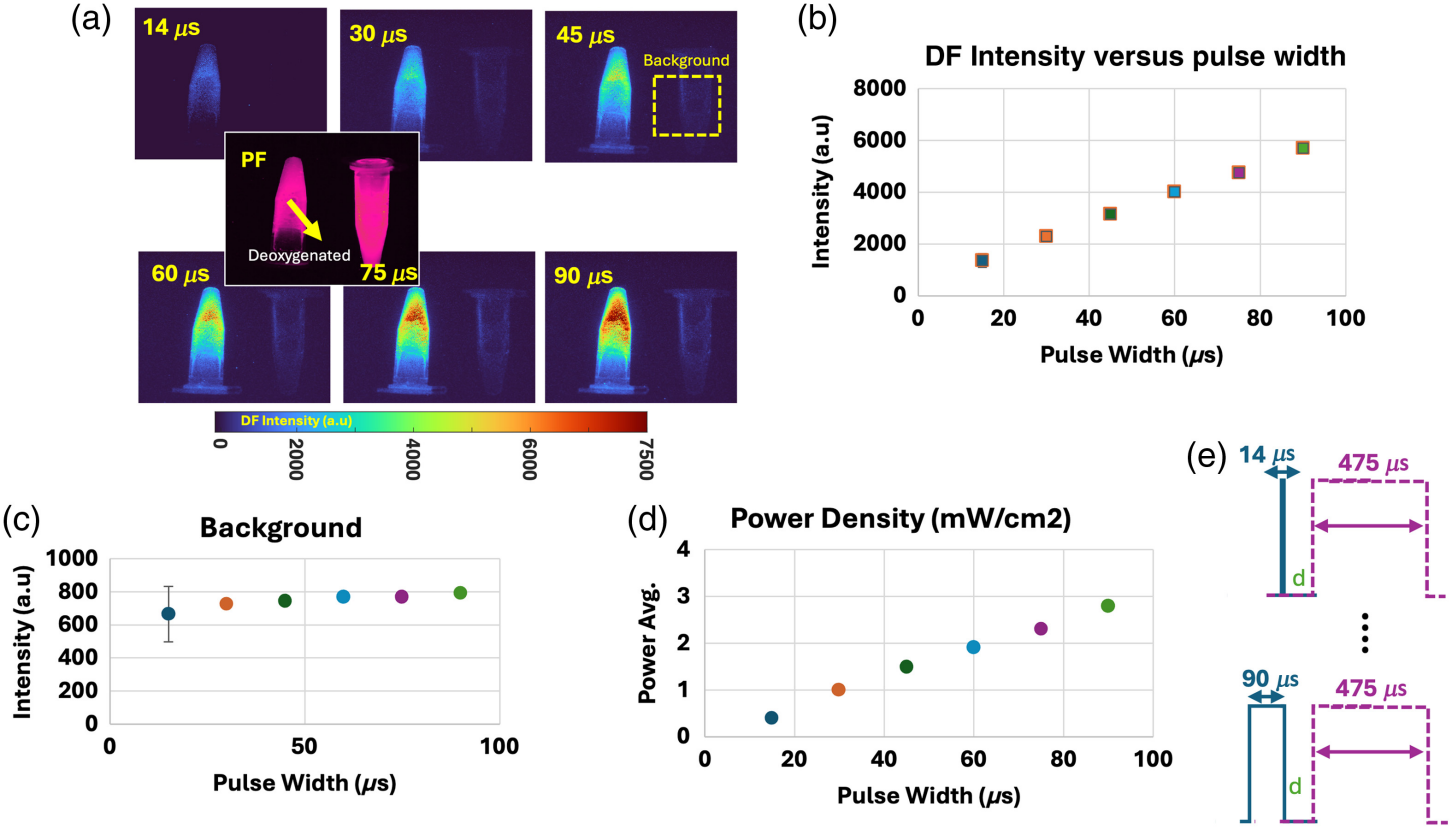
Effect of pulse-width increase and constant gate width. (a) Representative DF frames per used pulse width. A PF frame is shown as an example for 75  μs. PF frames are acquired during the excitation pulse, whereas DF frames are acquired by time gating after the excitation pulse/pulse width. (b) DF intensity as a function of pulse width when keeping a fixed gate width. (c) Recorded background intensity. (d) Power density ($\mathrm{mW}/{\mathrm{cm}}^{2}$) as a function of pulse-width increase. (e) Schematic of first and last pulse-width and fixed gate-width settings.

To examine the effect of power density on the DF output signal, we used a frequency of 2 kHZ with a fixed gate width of 475  μs, as established in the prior test, and varied the power density values. Instead of switching to higher power illumination sources, which can be inaccessible, the power density per pulse was efficiently altered by adjusting the pulse width of the excitation pulse. The methods are described in detail in Sec. 2.2.4. Figure 4 displays the results for excitation pulse widths of 14, 30, 45, 60, 75, and 90  μs. The light source power density respectively changed to 0.4, 1.0, 1.5, 1.9, 2.3, and $2.8\;\mathrm{mW}/{\mathrm{cm}}^{2}$ as the pulse width increased. The DF intensity increased linearly with increasing pulse width, as shown in Figs. 4(b) and 4(d). In this test, a constant gate width of 475  μs was maintained to utilize the majority of the available period at 2 kHZ and understand the role of power density/pulse width alone. Figure 4(a) provides a single example frame of DF *in vitro* acquired at an excitation pulse. Figure 4(c) provides the quantification of the DF recorded background. The background values are not significantly different upon increase of the DF signal. Gate widths smaller than 475  μs are explored in the upcoming section. As a result of pulse-width/power density optimization, DF output increased by a factor of ∼4.40.

### 3.4 Results for Longer Excitation Pulse Widths and Shorter Gate Widths on Signal Amplification for *In Vitro* Samples

**Fig. 5:**
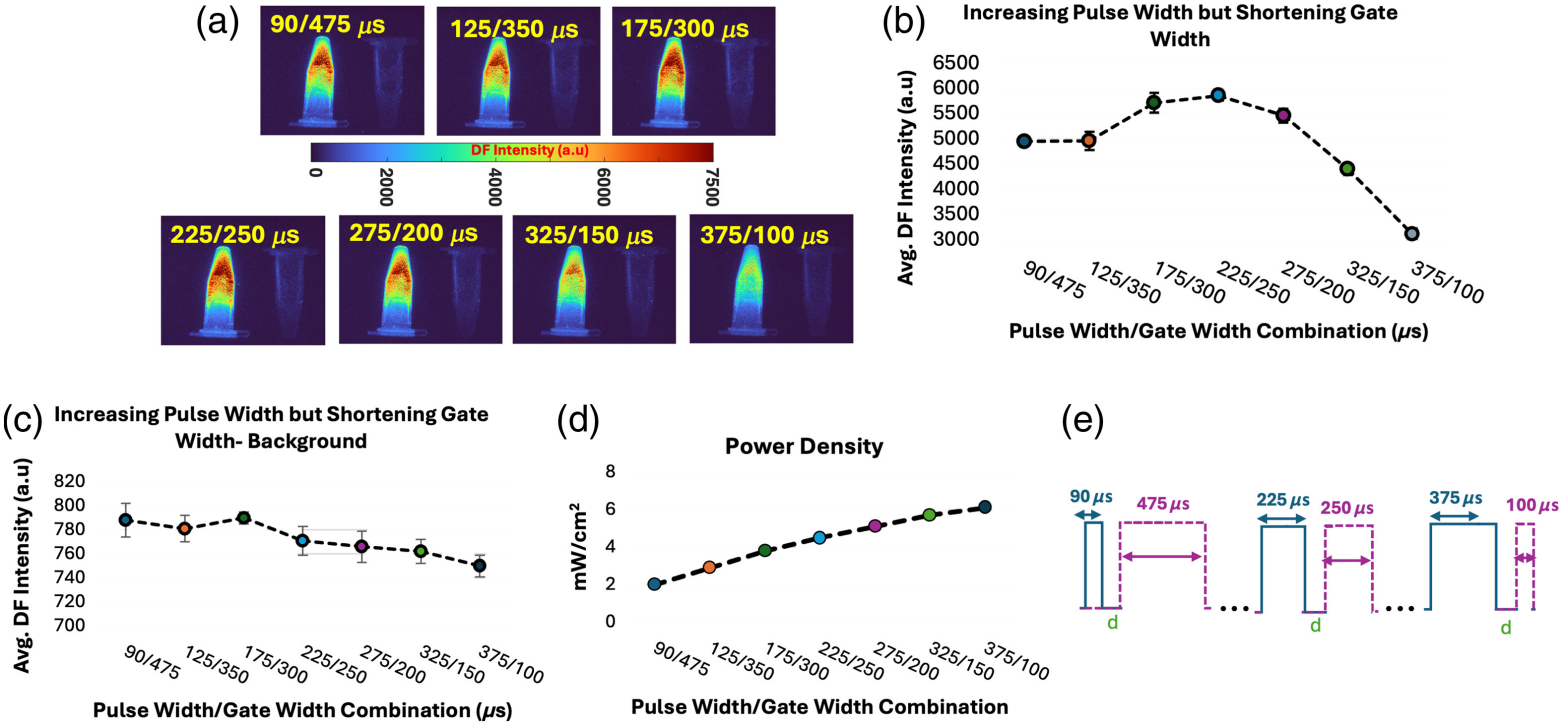
*In vitro* results for different combinations of pulse width and gate width. (a) Example of a single frame as acquired with each of the settings. (b) Quantification of intensity as a function of pulse-width/gate-width change. (c) Quantification of background as a function of pulse width/gate width. (d) Power density as a function of pulse width/gate width. (e) Schematic aid to understand pulse-width/gate-width distribution.

For a 2-kHZ pulse frequency or 500-μs periods, the previous test evaluated the effect of variable pulse-widths (ranging from 14 to 90  μs), collected with a constant gate-width of 475  μs. Although this gate width effectively collected close to the full period corresponding to a 2-kHZ frequency, the use of an even shorter gate width was explored here. The goal of this test was to understand the importance of power density, which is linear to pulse width and DF intensity output, as shown in Sec. 3.3 (Fig. 4), and its relationship to the size of time gate used for collection of photons. The methods are described in detail in Sec. 2.2.5. An initial hypothesis is that the larger the gate width (even when the pulse width is short) will result in a higher number of collected photons. This was with the notion that collecting more of the full decay of PpIX DF [Fig. 2(c)] was needed. To test this, pulse width/gate width combinations were tested for values of 90/475, 125/350, 175/300, 225/250, 275/200, 325/150, and 375/100  μs. *In vitro* results are displayed in Fig. 5. Figure 5(d) shows that further increasing the pulse width from 90 to 375  μs resulted in a power density increase from 2 to $6\;\mathrm{mW}/{\mathrm{cm}}^{2}$. Importantly, the increase in gate-width resulted in a slight decrease in recorded background signal, as shown in Fig. 5(c). As the pulse width increased and gate width decreased, a threshold pulse-width/gate-width setting of 225/250  μs was reached, as shown in Fig. 5(b), with an ideal ratio reached from 175/300 to 275/200  μs. Values for 325/150 pulse width to gate width being only under 500 counts below a setting of 90/475  μs gave insight into the importance of how the power density and photon excitation are more critical than the gate width of collection, especially for DF of PpIX. Through these settings, the signal could be further increased by a factor of ∼1.3 per frame.

### 3.5 Results of Estimated Temporal Oversampling Settings for a Tourniquet *In Vivo* Model

**Fig. 6:**
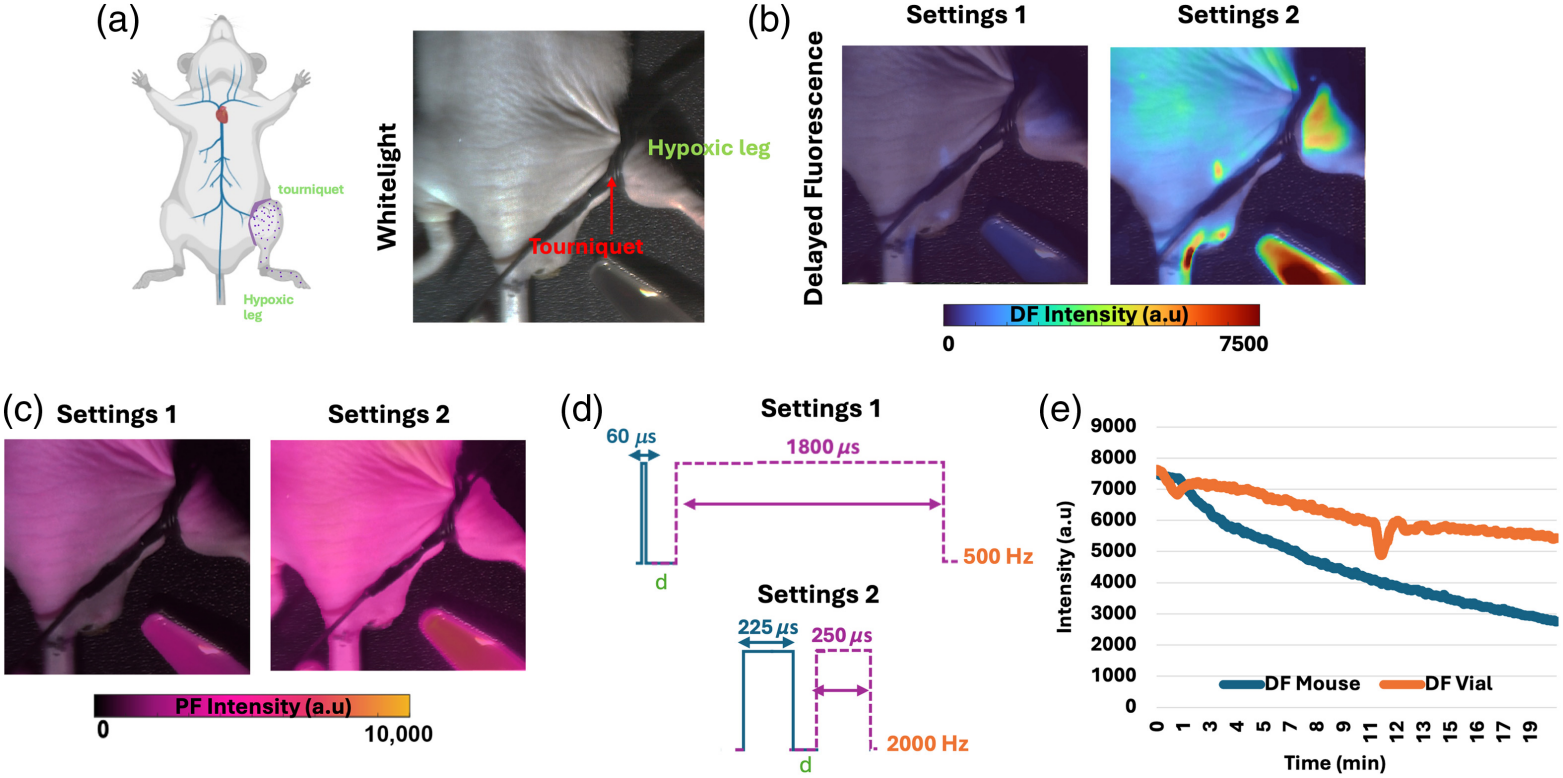
Results for the mouse model with induced hypoxia on the leg. (a) White light image of a mouse with a tourniquet on the right leg. Measure of (b) PpIX DF at settings 1 and 2 and (c) PpIX PF for settings 1 and 2. Deoxygenated control vials are placed next to the mouse for comparison. (d) Schematic of settings 1 (500 Hz) and settings 2 (2 kHz). (e) Average PpIX DF intensity *in vivo* compared with *in vitro* control over a period of 20 min. Set measured in a mouse (blue curve) *in vivo* and for PpIX control *in vitro* (orange curve).

The tests described in Secs. 3.2 to 3.4 were accomplished through *in vitro* models of oxygenated and deoxygenated PpIX solutions, where the level of deoxygenation was controlled. This section validated the optimal acquisition settings that were estimated *in vitro* for an *in vivo* tourniquet model. This *in vivo* model was also previously used in the FLS1000 and is depicted in Fig. 1 box 3. The method for tourniquet creation is described in Sec. 2.2.6. The results for 1 h after 5-ALA administration are displayed in Fig. 6. The results obtained on both DF and PF channels are respectively shown in Figs. 6(b) and 6(c). The results show that the DF of the hypoxic leg (only observed in DF channel) was increased by a factor of ∼6.5 when moving from settings that involved a short pulse width and long gate width (60/1800  μs) to a longer pulse width and shorter gate width (275/200  μs). Notably, even if the tourniquet was localized to the leg, it should be expected to see a DF signal increase across the surrounding skin as it is being pressed on. This pressure effect in the PpIX-based hypoxia signal has been previously reported.^11^^,^^12^ As power density was increased, PpIX photobleaching effects, which have been previously reported,^25^^,^^26^ need to be considered. To better understand photobleaching for this *in vivo* model as well as *in vitro*, a control vial was positioned in the FOV, and the mouse was continuously imaged for 20 min using the 2-kHZ settings. PpIX DF intensity values over time are displayed in Fig. 6(e), where after 20 min of continuous illumination, the *in vivo* signal intensity decreased by a factor of 2.6 compared with its initial value. This means that even for long-term exposure of 20 min, the factor gained through the proposed settings was approximately two times higher than what settings at 60  μs/500  Hz will yield in terms of recorded intensity. Although the intended 2.5-μM PpIX concentration in the vials was meant to match the *in vivo* intensity, we observed slower photobleaching of PpIX *in vitro* compared with *in vivo*, as shown in Fig. 6(e). Overall, even though photobleaching effects took place in this *in vivo* model, the usage of the 2-kHZ settings, as shown in Fig. 6(d), outweighed the amount of photobleaching happening over a period of 20 min.

### 3.6 Results of Estimated Temporal Oversampling Settings for AsPC1 Tumors *In Vivo*

**Fig. 7:**
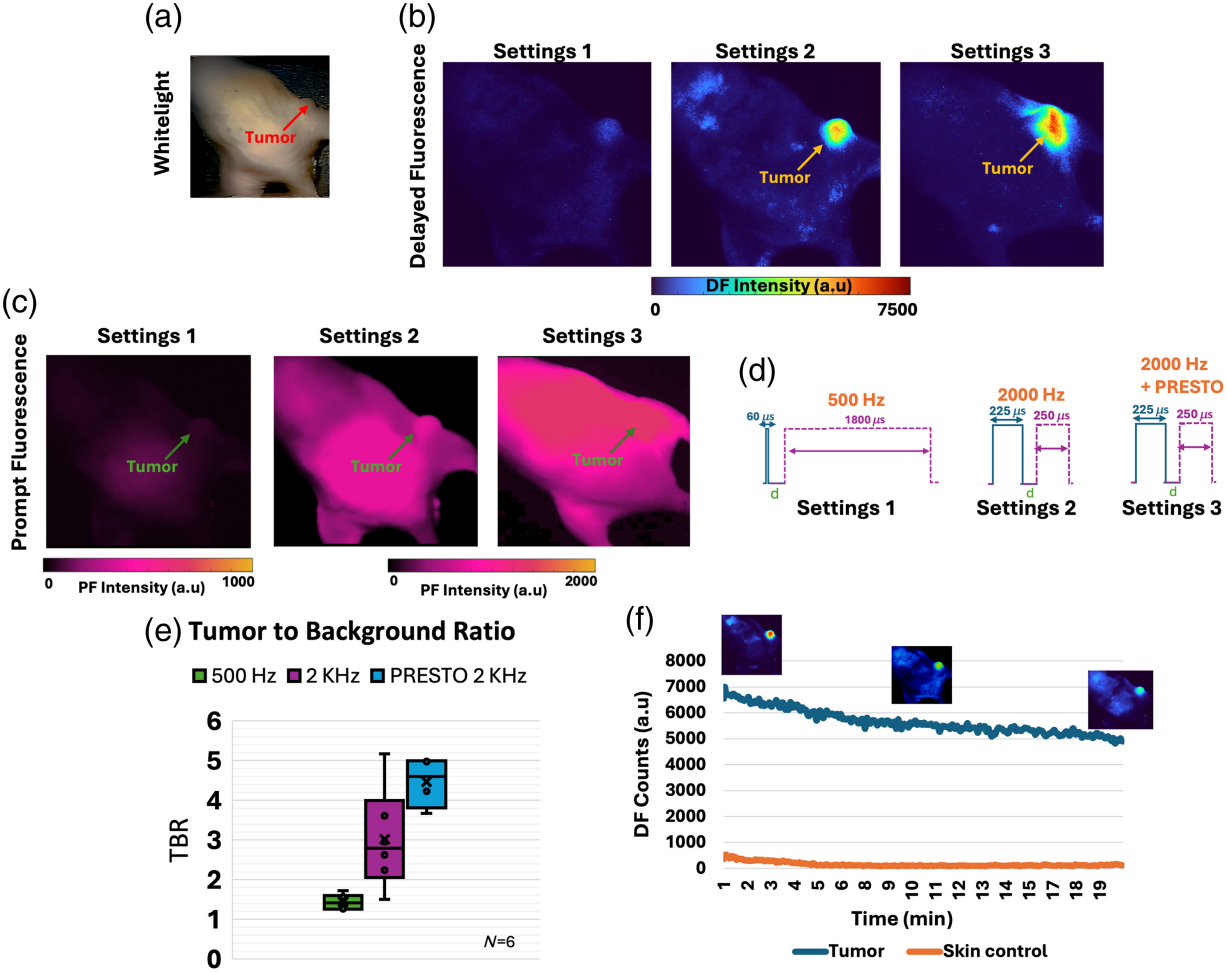
Results for the mouse model with AsPC1 tumor. (a) Whitelight image of a mouse with a tumor close to the right leg. (b) Measured PpIX DF for settings at 500 Hz (settings 1), 2 kHZ (settings 2), and 2 kHZ + PRESTO (settings 3) for hypoxia contrast. (c) PF of PpIX across the mouse FOV for the different settings. PF images illustrate how PpIX PF unlike PpIX DF is distributed across the mouse and not selective to the hypoxic tumor. (d) Schematic of settings used in column 1 (500 Hz), column 2 (2 kHZ), and column 3 (2 kHZ + PRESTO). (e) Quantification of signal-to-background ratio for a sample number of N=6. (f) DF intensity values as measured *in vivo* over a span of 20 min to quantify PpIX photobleaching effect in the tumor region. Skin is used as a control and compared with values when positioned over the tumor. Example DF frames are also displayed.

Finally, to validate whether the proposed settings consistently increased signal output across biological models, a mouse model of pancreatic adenocarcinoma (AsPC1) tumors was used. The method for AsPC1 tumor creation is described in Sec. 2.2.6. The results for mice imaged at 6 h post-5ALA injection are displayed in Fig. 7. As shown in Fig. 7(b), the DF channel successfully isolated the hypoxic tumor region. This could not be done through PpIX PF alone, as shown in Fig. 7(c). In addition, the tumor was pressed to create a transient hypoxia effect.^11^^,^^12^ Representative results are displayed in Fig. 7(b) for these three different scenarios. Figure 7(e) displays a quantification of tumor to background ratio (TBR) for a sample size of N=6, with the background chosen from the opposite leg. TBR values corresponding to the 500-Hz settings (1800-μs gate width) are below those recorded at 2 kHZ (250-μs gate width), as shown in Fig. 2(b). Furthermore, it was shown that pressing on the tumor created an additional TBR increase [see Fig. 7(e)] as previously reported.^11^^,^^12^ Hence, both techniques can be successfully employed in parallel to increase the PpIX DF signal output. Similar to Sec. 3.5, photobleaching effects in the tumor model were quantified, as in Fig. 7(f), during a ∼20-min exposure of the monitored tissue. In this case, photobleaching of the control skin area appears negligible in relation to photobleaching in the tumor area. Of note, the rate at which the tumor is photobleached was slower than the rate at which photobleaching happened in Fig. 6.

## 4 Discussion

The negligible differences between the spectrum of PpIX PF and DF [Fig. 2(b)] showed that the use of the same optical filter sets could be employed for retrieving both signals. However, as the DF lifetime component *in vivo* was ∼3133  μs, time gating after the excitation pulse retrieved only the DF of PpIX, which is representative of hypoxia as previously reported.^8^^,^^9^^,^^11^^,^^12^ Sampling the full lifetime decay of the PpIX DF signal as shown in Fig. 2(c) can be maximally beneficial to obtain an accurate ${\mathrm{pO}}_{2}$ estimation (through Stern–Volmer relationship);^27^ however, this comes at the cost of complex sampling and longer acquisition times, which can be unnecessary. In this case, if lifetime imaging and subsequent ${\mathrm{pO}}_{2}$ estimation are desired, the choice of a camera with a fast gate width to sample the lifetime decay is challenging. However, longer lifetimes such as PpIX DF [Fig. 2(c)] are accompanied by large increases in signal intensity, and so, sampling the DF intensity alone is an extremely efficient and effective way to achieve real-time hypoxia imaging.^11^^–^^13^^,^^28^ This is the core assumption going into this work, which is that sampling of the full lifetime was not necessary for practical imaging of hypoxia. Given this assumption, the signal could be further maximized by only sampling the emission in the earliest times after excitation, when the difference in DF signal intensity changes the most [see Fig. 2(c)]. This is accomplished through an increase in the sampling frequency, as demonstrated in Sec. 3.2, where shorter acquisition gates resulted in higher photon counts even when only sampling the first 475  μs of the DF emission decay. This is in comparison to a 500-Hz frequency where 1800  μs of the DF lifetime decay was sampled. The former clearly gains more photons and so has a stronger signal-to-background ratio. Sections 3.3 and 3.4 were devoted to examining and optimizing the pulse width to indicate the linearity of the DF signal output in relation to the increased power density. These sections explored the trade-off between having a long pulse width and constant gate-width window versus having a pulse width that is longer than the gate-width parameter. The results emphasized that if only the emission in the earliest times after excitation was sampled, a gate-width size of up to 200  μs at 2-kHz frequency could still provide a linear increase in PpIX DF signal. Sections 3.5 and 3.6 validated this hypothesis using *in vivo* models, for the best oversampling settings (2 kHz) retrieved from the *in vitro* testing. Photobleaching effects were observed due to the increase in power density. However, even when photobleaching was observed over a period of 20 min, the DF signal increase recorded using these optimized settings was still higher than that with the lower frequency settings with narrow pulse width and long gate width. Furthermore, the used power densities were below ranges used for commercial PpIX-based surgical guidance microscopes.^29^ Further *in vivo* studies are needed to fully examine the oxygen dependence of the photobleaching of PpIX as observed from DF, which could be correlated to the hypoxia seen *in vivo*. To date, this has been only investigated for PF of PpIX.^25^^,^^26^ Observed here, the photobleaching rate for tumors appeared to be slower than that displayed for the mouse tourniquet model. However, further validation is needed to correlate photobleaching kinetics to the response in hypoxia and possibly to the microlocalization of the PpIX *in vivo*. Throughout this work and for *in vivo* experiments, the observed decrease in intensity over a span of 20 min was mainly attributed to photobleaching. This was estimated as PpIX control vials also displayed this intensity decrease over time. An example trend for a control vial is plotted in Fig. 6(e) versus *in vivo* tissue. It is not expected for kinetics and dynamics to cause an intensity decrease in control vials as concentration and deoxygenation levels were controlled. However, it must be considered that in addition to photobleaching, for *in vivo* conditions, the kinetics and distribution dynamics of the tracer can also have an effect and in fact could explain why the decay rate is faster *in vivo* than *in vitro*. Future studies are needed to characterize the fundamental nature of the intensity decrease *in vivo*. A methodology to properly track PpIX concentrations across different organs *in vivo* for over 20 min would require a different optical configuration to allow for higher imaging depths and is beyond the scope of the current work.

## 5 Conclusions

This work explored the ability to mechanistically increase PpIX DF intensity, which is representative of tissue hypoxia, and boost its implementation for applications related to real-time imaging of metabolism and hypoxia events. This has been accomplished through an increase in sampling frequency, with the use of a shorter gate width and longer pulse width in the time domain. DF signal intensity was increased by an average of approximately sevenfold for *in vitro* and *in vivo* tests. Importantly, as represented in Figs. 6 and 7, this opens up the possibility to visualize additional hypoxic structures that might possess less intensity due to their size or anatomical location. Furthermore, when compared with settings that sample the full decay of PpIX DF but use a shorter excitation time, performance in target to background ratio was improved. In addition, this work demonstrated how these settings can be used in parallel to techniques such as PRESTO,^11^^,^^12^ which further increased the transient hypoxia DF signal in tissue. The effect of increased power density on photobleaching was also explored, demonstrating that even after 20 min of photobleaching, the hypoxia signal output remained higher than that observed with full-decay sampling settings. Future work could examine the idea of photobleaching rate as an additional parameter to understand hypoxia response *in vivo*. Even if full image exposure time settings were kept constant throughout this paper for comparison purposes, the used settings also increased the speed of acquisition as the frequency of sampling is increased. Further studies are needed to understand what is the minimum speed needed to resolve fast oxygen transients across anatomical structures. However, this is highly complex as it is dependent on the target structures and their oxygen kinetics, and the technique developed here can be used as a tool to study this.

## Data Availability

All data in the paper will be made available upon reasonable request to the primary authors. MATLAB algorithms can also be made available upon reasonable request.
